# Simultaneous Quantitative Analysis of the Major Bioactive Compounds in *Gentianae Radix* and its Beverages by UHPSFC–DAD

**DOI:** 10.1021/acs.jafc.2c01584

**Published:** 2022-06-13

**Authors:** Nora Gibitz-Eisath, Christoph Seger, Stefan Schwaiger, Sonja Sturm, Hermann Stuppner

**Affiliations:** † Institute of Pharmacy, Department of Pharmacognosy, CCB − Centrum of Chemistry and Biomedicine, CMBI - Center for Molecular Biosciences, University of Innsbruck, 6020 Innsbruck, Austria; ‡ Labordiagnostic St. Gallen West AG, 9015 St. Gallen, Switzerland

**Keywords:** yellow gentian, supercritical fluid chromatography, liqueurs, secoiridoids, natural product analysis

## Abstract

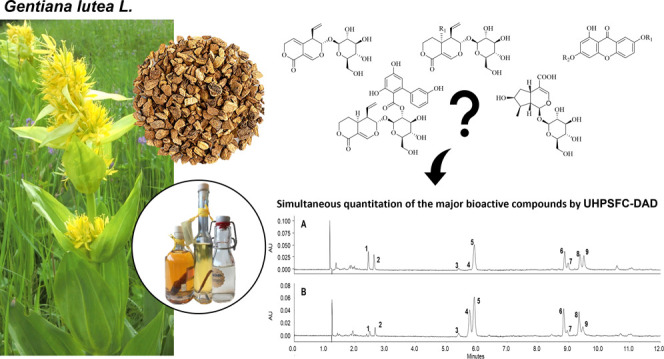

This study presents the first ultra-high performance supercritical fluid chromatography–diode array detector (UHPSFC–DAD) assay for simultaneous quantitation of secoiridoids, iridoids, xanthones, and xanthone glycosides in *Gentiana lutea* L. Separation was reached within 12 min on an Acquity UPC^2^ BEH 2-EP column using CO_2_ and methanol with 5.5% water as mobile phases. Method validation for nine selected marker compounds (gentisin, isogentisin, swertiamarin, sweroside, gentiopicroside, loganic acid, amarogentin, gentioside, and its isomer) confirmed the assay’s sensitivity, linearity, precision, and accuracy. The practical applicability was proven by the analysis of 13 root specimens and 10 commercial liquid preparations (seven liqueurs and three clear spirits). In all root batches, the secoiridoid gentiopicroside dominated (2.1–5.6%) clearly over all other metabolites. In the liqueurs, the metabolite content and distribution were extremely variable: while gentiopicroside was the main compound in four liqueurs, sweroside dominated in one preparation and loganic acid in two others. In contrast, measurable amounts of the metabolites were not detected in any of the examined clear spirits.

## 1 Introduction

Herbal preparations, especially those with bitter constituents, have taken on a firmly established role in the therapy of functional gastrointestinal diseases.^1^ Bitter constituents with their large chemical diversity belong to a waste number of different chemical compound classes and are distributed in many plant families.^2^ A widely used and well-studied plant in this context is yellow gentian (*Gentiana lutea* L., Gentianaceae). The medicinally used part of the plant is the root, which is listed in the European pharmacopeia (Ph. Eur.) under the monograph “*Gentianae Radix*”. It contains up to 8% bitter-tasting secoiridoid glycosides, including gentiopicroside as the main constituent, as well as amarogentin, swertiamarin, and sweroside.^3−6^ Due to the extensive use, the committee on herbal medicinal products (HMPC) declared the qualification as traditional herbal medicinal product as fulfilled and defined the indication “for temporary loss of appetite as well as for mild dyspeptic/gastrointestinal disorders” as supported.^7^ Similar statement is also given by the European Scientific Cooperative on Phytotherapy (ESCOP) who defined “loss of appetite e.g., after illness and dyspeptic complaints” as therapeutic indications.^8^ Beside the positive effect on the gastrointestinal tract, a variety of other pharmacological activities have been described for gentian root, including antioxidant,^9−11^ antimicrobial,^12^ antibacterial,^13,14^ antifungal,^15^ hepatoprotective,^16^ antiatherosclerotic,^17^ wound-healing,^18^ immunological,^19^ and secretolytic^20^ effects. These various effects are attributed not only to the contained bitter-tasting secoiridoid glycosides but also to further secondary metabolite classes.^11,12,18,21−23^ The xanthone isogentisin, for example, showed a cell-protecting activity, while its isomer gentisin was only recently identified as inhibitor of vascular smooth muscle cell proliferation.^25,26^

Beside its usage for medicinal purposes, in alpine regions, the root is also popular for the production of clear spirits and liqueurs. Both, clear spirits and liqueurs, are commercially available and used as appetizers and digestives since centuries. While clear spirits are obtained by the distillation of fermented gentian root mash, liqueurs are prepared by maceration: dried or fresh gentian roots are cut and steeped in spirit for several weeks.^27^ We wondered whether the substances known to mediate the aforementioned bioactivities can also be found in such preparations. Root samples of Ph. Eur. quality, the starting material for any pharmaceutical preparation but not necessarily for locally produced liqueur or clear spirit formulations, served as comparator samples.

An adequate quality assessment method for gentian root and preparations thereof must address all pharmacologically relevant compounds including bitter-tasting secoiridoids, iridoids, xanthones, and xanthone glycosides. However, even though several different analytical studies dealt with the quality control of *G. lutea* L., only a few studies published in the past two decades fulfilled this criterion.^28−32^ In addition, to the best of our knowledge, although gentian spirits and liqueurs are commercially available and widely used, quantitative investigations of these preparations are missing.

The fact that the published methods rely only on conventional HPLC-UV assays with total runtimes of at least 35 min encouraged us to search for a more innovative methodology for the rapid secondary metabolite profile assessment. We did choose ultra-high performance supercritical fluid chromatography (UHPSFC) for our analytical study since this interesting technique has shown big potential for natural product analysis and has never been used for this task before.^33−35^ Modern UHPSFC approaches are based on the combinatory use of carbon dioxide and organic solvents (so-called modifiers) as mobile phases. These mixtures are combining advantages like low viscosity and high diffusivity of gas chromatography with high dissolving capabilities and densities of liquid chromatography.^36^ Recent publications have not only demonstrated UHPSFC’s potential as an eco-friendly analytic alternative for highly polar plant constituents but also demonstrated its suitability for the simultaneous determination of compounds with divergent polarity, which makes it a powerful tool in the modern natural product analysis.^37−41^

## 2 Materials and Methods

### 2.1 Chemicals and Reference Compounds

All solvents and reagents (methanol, ethanol, isopropanol, formic acid) used in this study were of HPLC grade and purchased from Merck (Darmstadt, Germany). Carbon dioxide (4.5 grade, purity > 99.995%) was purchased from Messer (Gumpoldskirchen, Austria). Ultrapure water was produced by a Sartorius Arium 611 UV water purification system (Sartorius Stedim Biotech, Göttingen, Germany).

Thirteen samples of *G. radix* (R-1–R-13) were purchased from various companies in Austria and Germany. Voucher specimens (voucher nos. NGE-GL1–NGE-GL13) of all batches are deposited at the Institute of Pharmacy, University of Innsbruck. Three clear spirit samples (S-1–S-3) and seven traditionally prepared liqueur samples (L-1–L-7) were obtained from local suppliers (Austria and Italy). According to the suppliers, for the production of clear gentian spirits, the dried or fresh roots were fermented, and the obtained mash was distilled. For the liqueur preparation, the whole or cut roots were placed into a bottle, filled up with spirit, and kept under these conditions for at least 4 weeks.

The reference compounds swertiamarin **3**, sweroside **4**, gentiopicroside **5**, loganic acid **6**, and amarogentin **7** were obtained from PhytoLab (Vestenbergsreuth, Germany). Gentisin **1**, isogentisin **2**, gentioside **8**, and its isomer **9** were isolated from a methanol extract of *G. radix* (R-1) following a previously published protocol.^26^ Identity of the isolated reference compounds was confirmed by the analysis of spectroscopic and spectrometric data (1D- and 2D-NMR, LC-MS) and was in good agreement with the literature.^42^ Purity was determined by UHPSFC–DAD and was found to exceed 95% in all cases. NMR spectroscopic data and a detailed description of the isolation procedure of compounds **1**, **2**, **8**, and **9** are reported in the Supporting Information.

### 2.2 Sample Preparation

Gentian root batches (R-1–R-13) were finely pulverized to homogeneity with a coffee mill (3 × 1 min grinding time). The material (200.0 ± 0.1 mg) was weighed into 5.0 mL polyethylene microcentrifuge tubes (Eppendorf, Hamburg, Germany), mixed with 2.0 mL methanol on a Vortex mixer (VWR, Vienna, Austria), and sealed. Extraction was consequently conducted by sonication for 10 min at ambient temperature (Bandelin Sonorex, Berlin, Germany). To avoid temperature elevation, the water in the ultrasonic bath was changed after each extraction step. After the extraction, the samples were centrifuged at 10 600*g* for 5 min and the supernatant was transferred into a 10 mL volumetric flask. To ensure complete extraction (absolute recovery), the whole procedure was repeated four more times, the supernatants were combined, and the flask was filled up to volume with methanol.

Regarding the liquid preparations, 1.5 mL of each liqueur and clear spirit was evaporated to dryness and redissolved in 0.75 mL of MeOH. Recovery of this step was evaluated by the analogous workup of reference material samples dissolved in 40% EtOH/water (v/v). Due to the high concentration of **5**, samples L-3 and L-7 had to be diluted 1:1 (v/v) with methanol before quantitation. All sample solutions were prepared in triplicate, filtered (0.45 μm cellulose acetate membrane, VWR, Vienna, Austria), and stored at 4 °C until analysis.

### 2.3 UHPSFC–DAD and UHPSFC–MS Conditions

UHPSFC–DAD analysis was conducted on an Acquity UPC^2^ instrument, equipped with an autosampler, a binary solvent delivery pump, a column oven, a dual-stage active and static automated back-pressure regulator (ABPR), and a diode array detector (Waters, Milford, MA). An Acquity UPC^2^ BEH 2-EP column (3.0 × 150 mm, 1.7 μm particle size, Waters) was used as the stationary phase, and CO_2_ (A) and 5.5% water in methanol (B) were used as mobile phases. The gradient for separation was performed as follows: 100% A at 0 min, 85% A at 0.2 min, 85% A at 5.5 min, 83% A at 5.7 min, 79% A at 6.2 min, and 73% A at 10.0 min and held at this composition for 2 min (total runtime: 12 min); then, the column was equilibrated for 5 min under the initial conditions. Flow rate, column temperature, and ABPR were set to 1.10 mL/min, 35 °C, and 1500 psi. The injection volume was 1 μL, and the detection wavelengths were set to 232, 240, 257, and 268 nm.

UHPSFC–MS experiments for peak assignment and peak purity confirmation were performed by coupling the Acquity UPC^2^ system to a triple quadrupole mass spectrometer (Xevo TQD, Waters). UHPSFC separation conditions were identical to those described above. Methanol fortified with 0.5% formic acid was added as makeup solvent with a flow rate of 0.45 mL/min by a 515 pump and a pump control module (Waters). MS experiments were performed in the negative electrospray ionization (ESI) mode with the following parameters: desolvation temperature 250 °C, desolvation gas flow 200 L/h, capillary voltage 3.50 kV, and cone voltage 20 V. The scan range was set to 200–700 *m*/*z* with a scan time of 0.2 s.

### 2.4 Calibration and Method Validation

Two individual stock solutions of each standard compound (**1–9**) were prepared by separately weighing and dissolving them in methanol. Out of them, additional calibration levels were prepared by serial dilution. Calibration curves were obtained by plotting the peak areas versus the concentrations of the analytes. Linear regression analysis was used for the calculation of the regression parameters. Limit of detection (LOD) and limit of quantitation (LOQ) estimates were calculated from the regression models (including only the three lowest calibration levels) as 3 (LOD) or 10 times (LOQ) the residual standard deviation of the regression line divided by the slope. The lower LOQ was set to the lowest calibrator level.^43^ Precision was determined by triplicate analysis of three independently prepared *G. radix* samples of R-1 (intraday precision) on three consecutive days (interday precision) and is presented as the relative standard deviation (RSD) of the replicate measurements. Accuracy was determined at three different levels (low, medium, and high) by spiking sample R-1 with known amounts of standards **1** and **3–8** prior to sample workup. All samples were prepared in triplicate. To assess sample stability over the validation period, calibrators and samples of the first day were stored at 4 °C and remeasured on the last day. To address stability over the study period, a prealiquoted sample of R-1 was measured daily throughout the measurement campaign. In both cases, the relative deviation from the first measurement value was evaluated.

### 2.5 Statistics

Microsoft Excel 2016 (Redmond, WA) was used for the calculation of analyte concentrations and data analysis for validation.

## 3 Results and Discussion

### 3.1 Sample Preparation for UHPSFC Measurements

**Figure 1:**
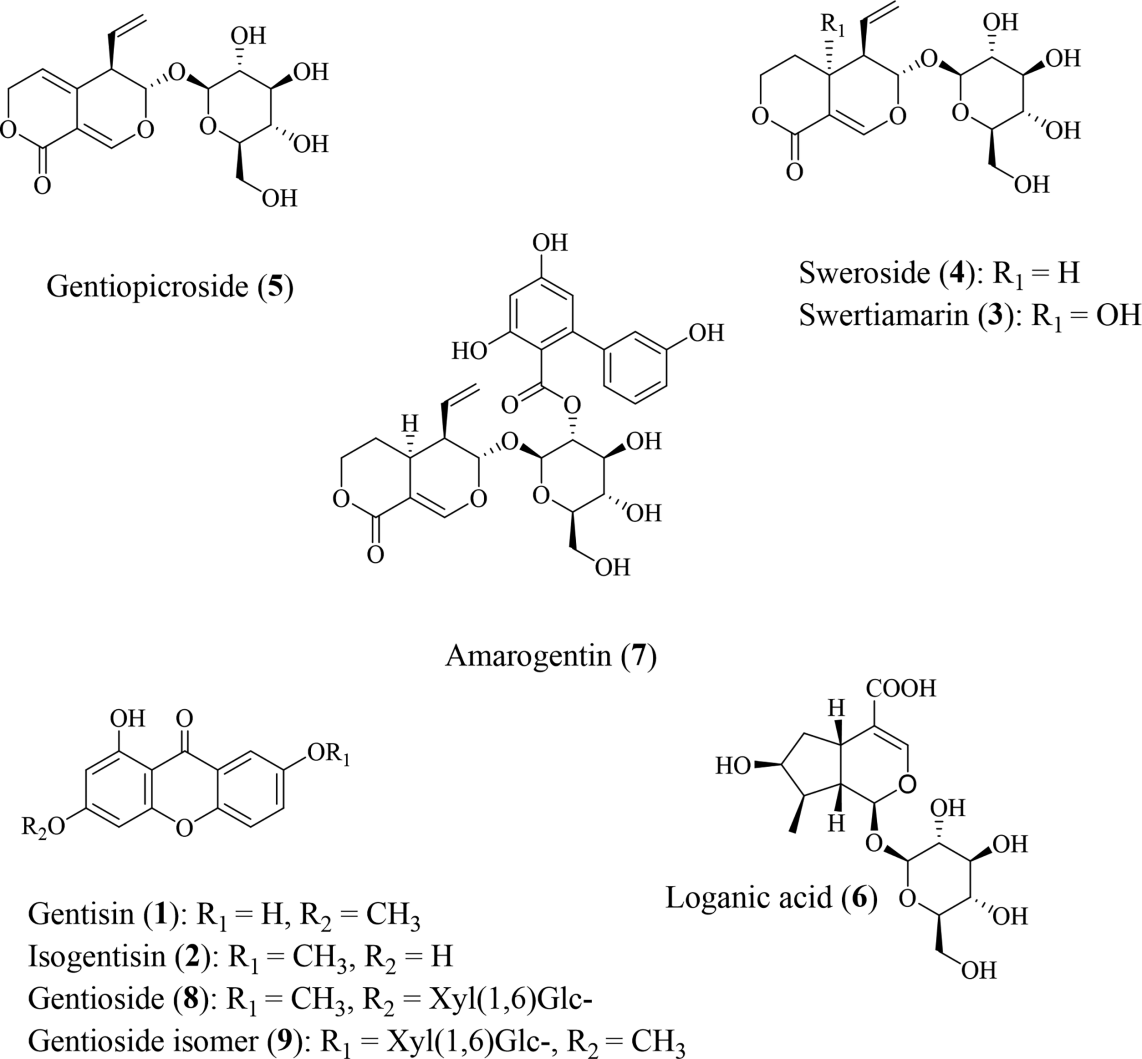
Chemical structures of the determined secondary metabolites in *G. radix*.

Two different sample types, namely, gentian root specimens and liquid beverages thereof, were investigated in this study. In the case of the root samples, the extraction procedure was adopted with slight modifications from a published protocol.^28^ Five cycles of ultrasound-assisted extraction with methanol as solvent turned out to be necessary for the exhaustive extraction of all relevant compounds, including secoiridoids, as well as iridoids, xanthones, and xanthone glycosides (see Figure 1 for structures). The suitability of the selected sample preparation procedure was proven as follows: a sample was extracted as described in Section 2.2. Then, the remaining root material was extracted once more, and the obtained supernatant was analyzed by UHPSFC. As in this solution, no quantifiable amounts of compounds **1–9** were found, the applied extraction protocol was considered exhaustive and suitable for quantitative investigation.

Several publications have highlighted the influence of sample solvent composition on peak shape and thus quantitative results in SFC separations.^44^ Therefore, to ensure a meaningful comparability of the quantitative results of root and beverage samples, clear spirits and liqueurs were not injected directly but evaporated to dryness and subsequently dissolved in methanol. To evaluate the possible analyte loss in the evaporation step, samples of analyte standards were prepared in a matrix mimicking the liqueurs and spirits and processed analogous to the liquid samples. Recoveries were found to be within the range of the validation experiments.

### 3.2 UHPSFC–DAD and UHPSFC–MS Method Developments

A methanolic gentian root extract and a standard mixture containing nine key metabolites (gentisin (**1**), isogentisin (**2**), swertiamarin (**3**), sweroside (**4**), gentiopicroside (**5**), loganic acid (**6**), amarogentin (**7**), gentioside (**8**), and its isomer (**9**); for structures, see Figure 1) served as samples for method development. The major hurdle in that respect was related to the wide polarity range of the analytes, on one side, and the close structural resemblance of some compounds, on the other side. Therefore, careful evaluation of all relevant separation parameters was necessary.

Supercritical CO_2_ is a highly lipophilic solvent with a polarity similar to hydrocarbons. Analysis of more polar compounds demands the addition of an organic solvent, the so-called modifier, to the mobile phase.^36^ Consequently, as first step, the performance of various modifiers (methanol, acetonitrile, isopropanol, and mixtures thereof) on different stationary phases (C-18, fluorophenyl, BEH 2-EP, DIOL, BEH) was evaluated. These experiments revealed that a mixture of CO_2_ and methanol as mobile phases and an Acquity UPC^2^ BEH 2-EP column are best suited for the described separation. It became obvious that in the SFC mode one had to face completely different separation challenges as in the HPLC mode. Aberham et al. described the separation of the xanthone aglyca gentisin **1** and isogentisin **2** as the major hurdle during the HPLC method optimization, reached only by changing the mobile phase to a mixture of acetonitrile and *n*-propanol.^28^ SFC mode, with its high orthogonality, showed to be perfectly suited for the separation of these apolar aglyca. However, several challenges were associated with the separation of the two polar xanthone glycosides **8** and **9**. Regardless of which solvent gradient and additive (formic acid, acetic acid, diethylamine, triethylamine) were used, no adequate peak shape and resolution could be obtained. Temperature, flow rate, and pressure modifications did not improve the results either. Several publications have underlined the suitability of water as an additive for SFC separations of highly polar compounds.^37,45−47^ Water tends to enhance the solvation power of the mobile phase leading to increased solubility of hydrophilic compounds, thus enabling faster elution and improved peak shapes.^48^ This trend was also observed in the current study. The addition of 5.5% water to methanol improved peak shapes significantly and enabled the adequate resolution of the xanthone glycosides.

In the present work, an Acquity UPC^2^ BEH 2-EP column with 5.5% water in methanol as a modifier, operating at a temperature of 35 °C, an ABPR set to 1500 psi, and a flow rate of 1.1 mL/min, was identified as optimal for the rapid separation (within 12 min) of all nine reference compounds. Due to the various UV maxima of the compounds of interest, different wavelengths were selected for their detection: 232 nm was chosen for the xanthones gentisin (**1**) and isogentisin (**2**), as well as for swertiamarin (**3**), loganic acid (**6**), and amarogentin (**7**), 240 nm for sweroside (**4**), 257 nm for the xanthone glycosides (**8, 9**), and 268 nm for gentiopicroside (**5**).

**Figure 2:**
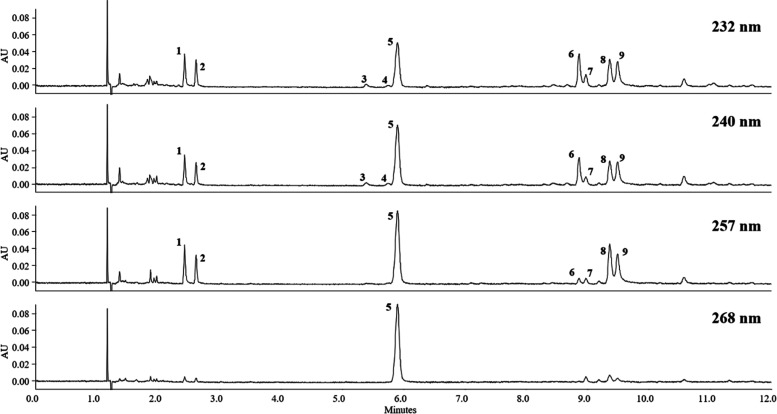
UHPSFC–DAD chromatograms of a *G. radix* extract (R-11) under optimized conditions: stationary phase: Acquity UPC^2^ BEH 2-EP (2.10 × 50.0 mm); mobile phase: CO_2_ (A) and MeOH with 5.5% H_2_O (B); gradient: 100% A at 0 min, 85% A at 0.2 min, 85% A at 5.5 min, 83% A at 5.7 min, 79% A at 6.2 min, and 73% A at 10.0 min, and held at this composition for 2 min; flow rate: 1.1 mL/min; temperature: 35 °C; ABPR: 1500 psi; and injection volume: 1 μL. Peak assignment is in accordance with Figure 1.

Subsequently, all nine compounds could be assigned in the *G. radix* extracts (Figure 2) by comparison of retention times, UV spectra, and spiking experiments. Peak assignment and peak purity were further confirmed by coupling the UHPSFC system to a triple quadrupole mass spectrometer via an electrospray ionization interface. Due to the physical nature and compressibility of CO_2_ mobile phases, the hyphenation of SFC to MS is not as simple as in LC but requires the addition of an organic solvent, the so-called makeup solvent via an interface, located ahead of the MS detector.^49,50^ In the current investigation, methanol fortified with 0.5% formic acid was identified as the best-suited makeup solvent, allowing adequate ionization of all compounds in the negative ionization mode. The obtained MS spectra were highly consistent within each peak and showed no evidence of coeluting compounds.

### 3.3 Validation of the UHPSFC–DAD Assay

To prove the suitability of the developed UHPSFC–DAD assay for quantitation purposes of compounds **1–9**, validation was performed according to the ICH guidelines.^43^ As shown in Table 1, calibration curves were linear over the tested concentration range with correlation coefficients (*R*^2^) always higher than 0.9991. LOD estimates ranged from 0.2 to 1.6 μg/mL, whereas the LOQ estimates were found to vary between 0.7 and 4.9 μg/mL. These values are significantly higher, compared to those of the HPLC-UV assay of Aberham et al. (LOD ≤ 37 ng/mL and LOQ ≤ 112 ng/mL).^28^ Regarding this observation, one has to consider that only 1 μL was injected in our UHPSFC assay, while 10 μL was injected in the HPLC assay. Another possible explanation is the often-criticized lower sensitivity of SFC-UV compared to that of HPLC-UV. This was observed, e.g., also by Dispas et al., who reported a 10-time lower sensitivity of SFC compared to that of LC using the same detector.^51^

**Table 1:** Calibration Data of the *G. lutea* UHPSFC–DAD Assay for Compounds **1–9**, Including Regression Equations, Correlation Coefficients (*R*^2^), and Linearity Range from LLOQ to ULOQ, LOD, and LOQ

compound	regression equation	*R*^2^	linearity range (μg/mL)	LOD (μg/mL)	LOQ (μg/mL)
**1**	*y* = 3573*x* – 4569.2	0.9993	7.8–250	0.2	0.7
**2**	*y* = 3316.6*x* – 12856	0.9992	3.7–570	0.5	1.6
**3**	*y* = 1026.9*x* – 1903.2	0.9999	7.7–1030	1.0	3.2
**4**	*y* = 1076.6*x* – 1307.8	0.9992	7.8–1050	0.7	2.1
**5**	*y* = 845.29*x* – 1800.3	0.9994	8.3–1000	1.6	4.9
**6**	*y* = 1044.2*x* + 2692.5	0.9993	9.1–1010	1.1	3.4
**7**	*y* = 2002.7*x* – 1452.5	0.9995	7.8–1150	0.8	2.6
**8**	*y* = 1856.1*x* + 3006.6	0.9991	6.6–105	0.6	1.9
**9**	*y* = 2053.2*x* + 5348.7	0.9991	12.4–198	1.3	3.9

Precision was assayed by the preparation and analysis of *G. radix* extracts (R-1) on three consecutive days. During method validation, both intraday (RSD ≤ 5.1%) and interday (RSD ≤ 3.6%) results were satisfying and in a normal range for plant analysis (Table 2). For the assessment of accuracy and extraction efficiency, the powdered root material was spiked with known amounts of the reference compounds **1** and **3–8** at three different concentration levels before sample workup. Recovery rates for all investigated compounds were in the accepted range with values between 96.7 and 107.7% (see Table 3). Sample stability over the time range of the project was found to be within the limitations of the assay precision.

**Table 2:** *G. lutea* UHPSFC–DAD Assay Precision for Compounds **1–9**^a^

compound	RSD (%)			
	day 1	day 2	day 3	days 1–3
**1**	2.4	2.3	2.7	2.6
**2**	2.2	4.0	3.7	3.3
**3**	3.2	2.9	3.0	3.6
**4**	2.0	1.5	2.7	2.1
**5**	1.7	0.7	1.9	1.5
**6**	2.6	5.1	3.1	3.6
**7**	2.4	2.5	2.6	2.7
**8**	4.5	1.5	3.0	3.4
**9**	1.4	3.2	2.4	2.7

a Intraday (*n* = 3 on each day) and interday (*n* = 9) precision.

**Table 3:** *G. lutea* UHPSFC–DAD Assay Accuracy for Compounds **1** and **3–8**^a^

compound	low spike		medium spike		high spike	
	added (μg/mL)	recovery (%)	added (μg/mL)	recovery (%)	added (μg/mL)	recovery (%)
**1**	5.0	104.8 ± 1.6	10.0	99.1 ± 1.4	15.0	96.0 ± 1.6
**3**	10.0	104.6 ± 6.6	15.0	100.9 ± 2.4	20.0	97.0 ± 1.7
**4**	10.0	98.7 ± 1.7	15.0	95.9 ± 2.4	20.0	99.6 ± 1.8
**5**	15.0	96.7 ± 6.5	70.0	99.5 ± 4.8	150.0	96.8 ± 3.6
**6**	10.0	96.9 ± 3.5	40.0	104.4 ± 2.4	80.0	102.4 ± 4.9
**7**	5.0	105.8 ± 2.3	10.0	100.0 ± 6.0	15.0	107.7 ± 4.9
**8**	10.0	102.9 ± 2.7	30.0	93.4 ± 2.6	40.0	102.6 ± 7.1

a Recovery values (*n* = 3) were expressed in the percentage of the amount added (mean ± relative standard).

### 3.4 Sample Analysis

**Figure 3:**
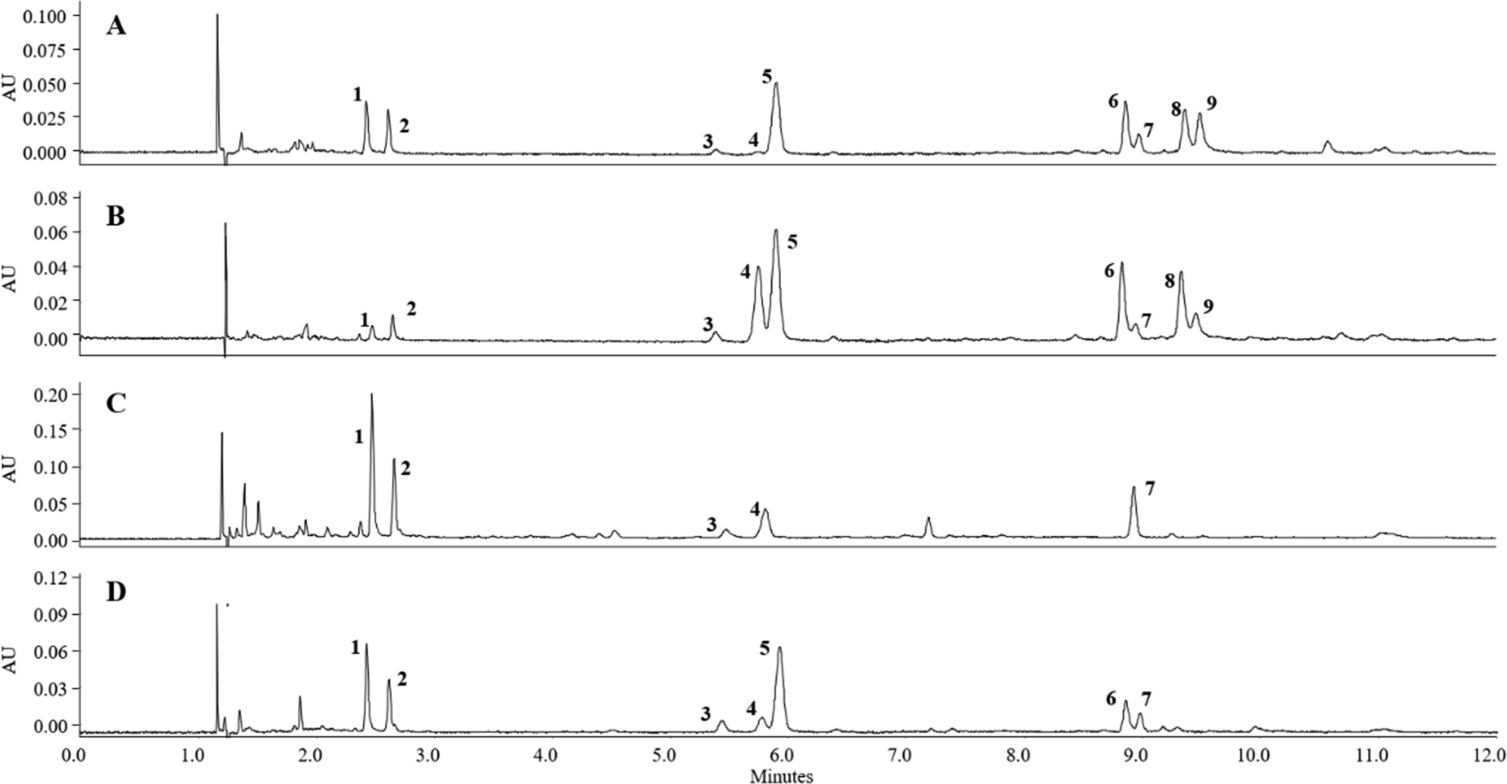
UHPSFC–DAD chromatograms of two gentian root samples (**A**: R-11 and **B**: R-8) and two liqueur preparations (**C**: L-4 and **D**: L-7) under optimized conditions. Detection wavelength: 232 nm. Peak assignment is in accordance with Figure 1.

The developed UHPSFC–DAD method was subsequently applied to the quantitative investigation of 13*G. radix* specimens, 7 liqueurs, and 3 clear spirits. Representative chromatograms of two *G. radix* extracts (R-11 and R-8) are shown in Figure 3A,B. The compiled quantitative results presented in Table 4 indicated that all nine reference compounds were detected in all investigated root specimens. The total content of bitter-tasting secoiridoids varied from 23.0 to 60.4 mg g^–1^ plant material, representing 2.3 to 6.0%, respectively. In all cases, gentiopicroside (**5**) was clearly the major compound with concentrations between 21.4 and 56.2 mg g^–1^ plant material. In 11 of the investigated 13 batches, the calculated gentiopicroside content reflected more than 90% of the total secoiridoid amount. All other secoiridoids (swertiamarin (**3**), sweroside (**4**), and amarogentin (**7**)) were present only in low concentrations. However, samples R-7 and R-8 were exceptions: the found sweroside (**4**) concentration of 13.1 and 10.8 mg g^–1^ plant material (representing 26.6 and 24.9% of the total secoiridoid content) was obviously higher compared to the other root samples, in some cases more than 10-fold. The concentration of amarogentin (**7**), the most bitter natural product known to date, was in all samples below 2.1 mg g^–1^ plant material. However, due to its high bitter value (58 million; in comparison: gentiopicroside: 12 000), it is anyway one of the value-determining ingredients.^3,5,6^

**Table 4:** Quantitative Results for Compounds **1–9** in Different *G. radix* Batches; All Values Are in mg g^–1^ Plant Material (with RSD in Parentheses; *n* = 3)

	**1**	**2**	**3**	**4**	**5**	**6**	**7**	**8**	**9**
**R-1**	0.47 (2.4)	0.69 (4.3)	1.47 (2.5)	1.07 (2.5)	27.15 (4.2)	6.70 (2.0)	0.26 (4.3)	4.86 (4.5)	3.37 (2.9)
**R-2**	0.92 (3.4)	1.33 (2.1)	1.19 (2.2)	0.82 (1.1)	28.63 (2.1)	5.17 (1.2)	<LOQ	4.39 (2.2)	3.11 (1.5)
**R-3**	0.87 (2.1)	0.84 (1.1)	2.09 (1.9)	0.44 (1.3)	39.21 (0.8)	5.12 (1.2)	0.14 (5.6)	5.37 (2.4)	4.46 (1.7)
**R-4**	1.43 (2.2)	2.49 (1.4)	0.72 (1.6)	0.37 (4.0)	21.37 (2.0)	1.11 (4.2)	0.55 (2.0)	1.58 (2.1)	0.28 (4.9)
**R-5**	0.77 (1.6)	1.11 (0.8)	1.77 (4.2)	0.76 (2.7)	30.87 (2.1)	4.50 (1.2)	<LOQ	3.24 (7.8)	2.18 (2.0)
**R-6**	0.65 (1.4)	0.79 (1.2)	0.97 (2.0)	1.15 (3.6)	24.45 (1.6)	8.00 (1.2)	0.38 (1.9)	7.30 (1.4)	5.83 (2.0)
**R-7**	0.49 (2.6)	1.00 (1.7)	1.49 (2.9)	13.09 (0.8)	32.57 (0.4)	1.02 (3.7)	2.11 (0.9)	3.32 (1.7)	4.64 (0.5)
**R-8**	0.15 (2.8)	0.36 (1.4)	1.27 (2.5)	10.83 (2.0)	30.94 (1.0)	7.73 (1.7)	0.47 (2.2)	5.69 (2.7)	2.06 (1.1)
**R-9**	0.86 (2.1)	1.30 (3.3)	1.06 (1.7)	1.16 (1.7)	27.91 (3.6)	5.72 (3.6)	<LOQ	3.83 (2.8)	2.88 (3.1)
**R-10**	0.78 (2.6)	0.88 (1.1)	2.54 (2.2)	1.54 (1.5)	56.17 (2.1)	5.69 (1.9)	0.15 (2.9)	2.76 (0.9)	1.79 (1.7)
**R-11**	1.11 (3.8)	1.01 (3.8)	1.02 (3.4)	0.59 (1.2)	27.38 (2.0)	6.48 (1.9)	1.02 (3.8)	4.93 (2.2)	3.96 (2.2)
**R-12**	0.73 (1.6)	0.77 (2.5)	2.52 (2.1)	1.77 (3.0)	53.37 (1.7)	5.97 (1.5)	<LOQ	2.69 (1.3)	1.70 (2.9)
**R-13**	1.07 (1.8)	1.08 (1.1)	1.03 (2.4)	0.52 (3.2)	27.82 (1.0)	5.80 (1.3)	1.02 (2.3)	5.49 (1.1)	4.32 (1.3)

Regarding the xanthones, it was observed that the glycosides (**8** and **9**) predominated clearly over the aglyca (**1** and **2**). In addition, it was noted that gentioside (**8**) and its corresponding aglycon (**2**) were dominating over the respective isomers in almost all samples. All of these observations were in good agreement with the already published data.^7,28,52^

In the alpine region, two different kinds of liquid gentian root preparations, namely, liqueurs and clear spirits, are commercially available and traditionally used to stimulate appetite and increase digestion.^27^ To investigate whether significant amounts of the main bioactive compounds are contained, different liquid preparations, obtained from different suppliers in Austria and northern Italy, were analyzed. Gentian liqueurs are produced by simple maceration: the dried or fresh gentian roots are cut and steeped for several weeks in spirit. However, it must be considered that the preparation is not standardized between different suppliers: neither the amount of root material per bottle nor the exact extraction time is known. Due to these facts, strong variations between different preparations were already expected. However, as can be seen from the compiled results shown in Table 5, the variations were even larger than expected. The gentiopicroside concentration (**5**) varied from 2.22 mg/L (sample L-1) to 905.1 mg/L (sample L-3). Gentiopicroside was the main compound only in four liqueurs (L-3, L-5, L-6, L-7). In one liqueur (L-4), with a concentration of 85.3 mg/L, sweroside (**4**) was identified as the major compound, while in other two (L-1, L-2), loganic acid (**6**) dominated. These findings were quite unexpected, as in all investigated root samples (Table 4) gentiopicroside (**5**) was clearly dominating over all other quantified metabolites. As reported in the literature, the intense yellow color of the liqueurs is attributed to the contained xanthone derivatives.^7^ However, as displayed in representative chromatograms (Figure 3C,D), only the two aglyca gentisin (**1**) and isogentin (**2**) were detected. The respective glycosides **8** and **9**, which occurred in quite high concentrations in the root batches (see Table 4 and Figure 3A,B), were not present in any investigated liqueur preparation.

**Table 5:** Quantitative Results for Compounds **1–9** in Different *G. radix* Liqueur Samples; All Values Are in mg L^–1^ Liqueur (with RSD in Parentheses; *n* = 3)

	**1**	**2**	**3**	**4**	**5**	**6**	**7**	**8**	**9**
**L-1**	3.55 (1.5)	1.85 (3.8)	<LOQ	9.30 (2.1)	2.22 (4.1)	35.54 (4.0)	5.14 (3.9)	<LOQ	<LOQ
**L-2**	29.44 (2.6)	41.88 (2.5)	44.68 (1.3)	13.92 (2.6)	4.01 (2.3)	181.78 (2.9)	7.52 (2.0)	<LOQ	<LOQ
**L-3**	10.05 (2.2)	14.62 (1.4)	18.20 (2.1)	57.52 (1.4)	905.10 (1.5)	68.06 (1.4)	13.28 (1.6)	<LOQ	<LOQ
**L-4**	38.43 (1.8)	16.98 (1.3)	30.84 (1.5)	85.31 (2.1)	4.45 (4.3)	<LOQ	49.64 (1.9)	<LOQ	<LOQ
**L-5**	65.29 (1.5)	32.68 (1.8)	9.75 (1.7)	42.59 (1.0)	66.06 (1.2)	13.27 (2.4)	27.02 (1.6)	<LOQ	<LOQ
**L-6**	13.46 (1.5)	6.87 (2.7)	13.97 (1.9)	4.09 (3.6)	56.53 (1.5)	28.42 (2.4)	3.55 (3.9)	<LOQ	<LOQ
**L-7**	34.73 (3.9)	25.68 (4.9)	40.21 (1.8)	45.87 (1.8)	661.31 (3.0)	78.84 (2.0)	18.97 (2.0)	<LOQ	<LOQ

In contrast to gentian liqueurs, clear gentian spirits are obtained by distillation of the fermented root mash.^27^ Three different clear spirits were available for investigation. Measurable contents of the chosen reference compounds (**1–9**) were not observed in any of these. Due to the manufacturing process and the nonvolatile character of the investigated compounds (see Figure 1 for structures), these results were unsurprising.

Taken together, the results are underlining the excellent suitability of the UHPSFC assay for the quality control of gentian, combining features of rapid and highly efficient separation performance with advantages of low operating costs and eco-friendliness. The fact that the assay enables the simultaneous determination of polar secondary metabolites with divergent characteristics, including bitter-tasting secoiridoids, as well as iridoids, xanthones, and xanthone glycosides, highlights once more the broad and still often underestimated potential of SFC for natural product analysis. With a total analysis time of 12 min, thus a reduction to one-third, the method represents a significant improvement and advantage compared to the conventional HPLC assay.^28^ We could show that beside the quality control of gentian root batches, the method is additionally a useful and reliable tool for the investigation of commercially available liqueurs and spirits. However, as shown by the presented results, the content of the pharmacologically relevant secondary metabolites in liquid preparations is varying strongly. To explain the reasons for these variations, further investigations with a bigger sample set, as well as with profound knowledge of the origin of the root material used for production and the exact preparation conditions, are needed. Generally, the rich secondary metabolite profile of the root materials of pharmacopeia quality cannot be found in the fermentation or maceration-based liquids. Hence, the health benefits of liqueur/spirit consumption cannot be expected to meet the benefits associated with the use of Ph. Eur. quality material.

## Supporting Information Available

NMR spectroscopic data and a detailed description of the isolation procedure of the xanthones gentisin **1** and isogentisin **2** and the xanthone glycosides gentioside **8** (= isogentisin-3-*O*-primveroside) and gentioside-isomer **9** (= gentisin-7-*O*-primveroside) (PDF)

## Abbreviations

ABPR: automated back-pressure regulator
CO_2_: carbon dioxide
DAD: diode array detector
LOD: limit of detection
LOQ: limit of quantitation
MeOH: methanol
UHPSFC: ultra-high performance supercritical fluid chromatography
