# Supplementary material for: Simultaneous Quantitative Analysis of the Major Bioactive Compounds in Gentianae Radix and its Beverages by UHPSFC–DAD
Source: J Agric Food Chem. 2022 Jun 13;70(24):7586–93. doi: 10.1021/acs.jafc.2c01584 (PMC9228070; doi:10.1021/acs.jafc.2c01584)
Supplement: Supplementary file 1 — jf2c01584_si_001.pdf [file jf2c01584_si_001.pdf]

SUPPLEMENTARY MATERIALS

Simultaneous quantitative analysis of the major bioactive compounds in *Gentianae radix* and its beverages by UHPSFC-DAD

Nora Gibitz-Eisath[1,2], Christoph Seger[1,2], Stefan Schwaiger[1], Sonja Sturm[1*], Hermann Stuppner[1]

Affiliation

[1] Institute of Pharmacy, Department of Pharmacognosy, CCB – Centrum of Chemistry and Biomedicine, CMBI - Center for Molecular Biosciences, University of Innsbruck, Innsbruck, Austria

[2] Labordiagnostic St. Gallen West AG, St. Gallen, Switzerland

***Correspondence**

Dr. Sonja Sturm, Institute of Pharmacy, CCB – Centrum of Chemistry and Biomedicine, University of Innsbruck, Department of Pharmacognosy, Innrain 80/82, 6020 Innsbruck, Austria. Phone: +4351250758408 Fax: +4351250758499 e-mail: sonja.sturm@uibk.ac.at

Isolation of xanthones and xanthone glycosides

Powdered gentian root material (R-10, 500g) was extracted with methanol by maceration yielding 238 g crude extract. The crude extract was dissolved in 1000 ml water and fractionated by liquid–liquid distribution with different organic solvents (petroleum ether, diethyl ether, ethyl acetate, 1-butanol; five times, each 500 ml). The resulting organic fractions were evaporated to dryness yielding 20.2 g petroleum ether fraction, 26.8 g diethyl ether fraction, 6.2 g ethyl acetate fraction, 40.2 g 1-butanol fraction, and 146.4 g water fraction.

The diethyl ether fraction (26.8 g) was chromatographed on silica gel (400 g) employing a gradient of dichloromethane and ethyl acetate (from 100:0 to 0:100, v/v), yielding 12 fractions (A1-A12). 180.2 mg of fraction A6 (443.9 mg) was purified by recrystallization from hot methanol, yielding 56.0 mg gentisin (**1**). Fraction A5 (450 mg) was separated by flash silica column chromatography (Reveleris, Büchi, Flawil, Switzerland) using a gradient with dichloromethane and ethylacetate, resulting in the isolation of 54.1 mg isogentisin (**2**).

The ethyl acetate fraction (6.2 g) was suspended in 10 ml methanol and centrifuged at 3000 rpm for 10 minutes (Labofuge 400, Heraeus, Hanau, Germany). The supernatant was discharged and the solid residue was anew suspended in methanol and centrifuged. This procedure was repeated eight more times. The obtained solid residue (129 mg) contained a mixture of both, gentioside and its isomere and was dissolved in DMSO/water 1:1 (22 mg/mL) and separated by semi preparative HPLC on an UlitiMate 3000 preparative HPLC system (Dionex-Thermo Inc., Waltham, MA, USA). Optimum separation was carried out on a Synegri MAX RP column (250 mm x 10 mm, 4.0 µm) using an isocratic gradient of 75% water and 25% acetonitrile over 45 min. Flow rate, temperature, injection volume, and detection wavelength were set to 1.8 mL/min, 40°C, 20 µL, and 230 nm. 14.3 mg gentioside (**8**) and 9.4 mg of its isomere (**9**) could be isolated.

Identity was confirmed by LC-MS and 1D and 2D-NMR (see Appendix Table 3) experiments as well as comparison with published data. The NMR data, obtained on a Bruker Avance II 600 spectrometer (Karlsruhe, Germany), operating at 300 K, is reported in the following tables.

NMR spectroscopic data (600.19 MHz (δ_H)/150.91 MHz(δ_C)) of the isolated compounds

Supplementary Table 1. NMR spectroscopic data (600.19 MHz (δ_H)/150.91 MHz (δ_C)) of the xanthones gentisin **1** and isogentisin **2**.

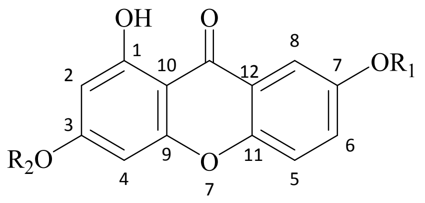

Gentisin: $R_1 = H$, $R_2 = CH_3$
Isogentisin: $R_1 = CH_3$, $R_2 = H$

**Position**	**Gentisin (DMSO-*d*$_6$)**		**Isogentisin (MeOH-*d*$_4$)**	
	**δ_C**	**δ_H (*J* in Hz)**	**δ_C**	**δ_H (*J* in Hz)**
**1**	162.5	-	164.7	-
**2**	96.9	6.40, d (2.3)	99.2	6.19, d (2.1)
**3**	166.4	-	167.7	-
**4**	92.6	6.63, d (2.2)	95.0	6.33, d (2.1)
**5**	119.0	7.51, d (9.0)	120.1	7.44, d (9.1)
**6**	124.8	7.33, dd (9.0, 3.0)	125.5	7.37, dd (9.1, 3.1)
**7**	154.1	-	159.5	-
**8**	108.0	7.44, d (3.1)	106.5	7.60, d (3.1)
**9**	157.4	-	157.5	-
**10**	102.8	-	103.6	-
**11**	149.1	-	152.1	-
**12**	120.4	-	122.0	-
**C=O**	180.1	-	181.6	-
**O-CH_3**	56.1	3.90, s	56.3	3.90, s

Supplementary Table 2. NMR spectroscopic data (600.19 MHz (δ_H)/150.91 MHz (δ_C)) of the xanthone glycosides gentioside **8** (= isogentisin-3-*O*-primveroside) and gentioside-isomer **9** (= gentisin-7-*O*-primveroside).

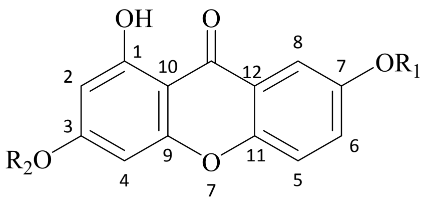

Gentioside: $R_1 = CH_3$, R_2 = Xyl(1,6)Glc
Gentiosideisomer: R_1 = Xyl(1,6)Glc, $R_2 = CH_3$

**Position**	**Gentioside (DMSO-*d₆*)**		**Gentioside-isomer (DMSO-*d₆*)**	
	**δ_C**	**δ_H (*J* in Hz)**	**δ_C**	**δ_H (*J* in Hz)**
**1**	162.2	-	162.61	-
**2**	98.7	6.47, br s	97.02	6.41, d (2.3)
**3**	164.2	-	166.55	-
**4**	94.6	6.77, br s	93.04	6.64, d (2.3)
**5**	119.5	7.66, d (9.0)	119.09	7.61, d (8.9)
**6**	124.9	7.49, dd (9.1, 3.2)	125.69	7.67, dd (9.0, 3.0)
**7**	155.8	-	153.82	-
**8**	105.3	7.54, d (3.1)	110.65	7.69, d (3.0)
**9**	157.2	-	157.35	-
**10**	103.5	-	102.88	-
**11**	150.3	-	150.76	-
**12**	120.2	-	120.34	-
**C=O**	180.0	-	179.81	-
**O-CH_3**	55.8	3.89, s	56.13	3.90, s
**1´´**	104.2	4.19, d (7.5)	103.98	4.18, d (7.3)
**1´**	99.8	5.04, d (7.1)	101.32	4.91, m
**3´´**	76.5	3.11, td (8.8, 3.5)	76.44	3.07, td (8.7, 4.2)
**2´/ 4´´**	76.3	3.30, m	76.23	3.30, m
**3'**	75.7	3.67, m	75.84	3.58, m
**2´´**	73.4	3.04, m	73.32	3.03, m
**2´/ 4´´**	73.0	3.29, m	73.19	3.29, m
**4´**	69.6	3.20, m	69.58	3.20, m
**5´**	69.5	3.34, m	69.53	3.29, m
**6´**	65.6	3.73, dd (11.3, 5.3)<br>3.03, m	65.56	3.68, dd (11.3, 5.4)<br>2.98, m
